# Injury epidemiology in professional football in South America compared with Europe

**DOI:** 10.1136/bmjsem-2021-001172

**Published:** 2021-10-01

**Authors:** Håkan Bengtsson, Pablo Alfredo Ortega Gallo, Jan Ekstrand

**Affiliations:** 1 Unit of Community Medicine, Department of Health, Medicine and Caring Sciences, Linköpings Universitet, Linköping, Östergötland, Sweden; 2 Football Research Group, Linköpings Universitet, Linköping, Östergötland, Sweden; 3 School of Medicine, Pontificia Universidad Catolica Argentina, Buenos Aires, Argentina

**Keywords:** football, epidemiology, injury

## Abstract

**Objective:**

To describe the injury epidemiology in professional football in South America and compare it with European professional football.

**Methods:**

Data about football exposures and injury occurrences were registered in Six teams participating in Copa Libertadores in 2016. These teams’ exposure and injury data were compared with teams participating in the UEFA Elite Club Injury Study during the 2015/2016 and 2016/2017 seasons.

**Results:**

A total of 271 injuries were reported in the South American cohort representing a training injury incidence of 3.2 (95% CI=2.7 to 3.7) injuries/1000 hours of training exposure and 20.9 (95% CI=17.3 to 25.1) injuries/1000 hours of match exposure. While no differences in muscle injury incidence were observed between South American and European teams, the ligament injury incidence in training among South American teams was significantly higher than European teams (0.6 vs 0.3, RR 1.87, 95% CI 1.21 to 2.87). In addition, a significantly higher proportion of all reported injuries among South American teams than European teams occurred in training.

**Conclusions:**

A larger proportion of injuries occur in training in South American compared with European professional football. Specifically, ligament injuries in training were more frequent among South American teams.

**Key messages**

**What is already known?**

- Even though multiple studies have shown that injuries are common in professional football, most of this information stems from epidemiological studies conducted in Europe.
- Injury epidemiology may differ due to contextual factors, which could result in different injury patterns at other regions.

**What are the new findings?**

- The training to match exposure ratio was higher among South American teams compared with European teams but the general injury epidemiology in training and match-play were similar.
- Ligament injuries in training were more frequent in South American teams than in European teams and caused a significantly higher injury burden, while muscle injury frequency was similar in both cohorts.

## Introduction

According to the international football association, FIFA, football is a global sport with more than 265 000 000 people directly involved.^1^ While several beneficial health effects could be reached through playing football,^2–4^ there is also a high risk of injury, especially for professional football players.^5^

The aetiology of sports injuries is complex, and there are several different risk factors, intrinsic and extrinsic, that could contribute to an athlete sustaining an injury.^6^ There are thus reasons to believe that injury epidemiology may differ between different regions and associations, for example, due to differences in climate, competition formats, etc.^7–9^ Most of the previously published studies analysing the risk of injury for professional football players have, however, been conducted in Europe. There is not as much information about the injury epidemiology in other geographic regions.^10^ In a review article from 2020, only two studies had investigated the injury epidemiology in South American football included and only one of these, including a team followed over one season, concerned professional club football.^11^ To describe the injury epidemiology in a sport is often considered the first fundamental step in injury prevention.^12^ The aim of this study was thus to analyse the injury epidemiology among South American male professional football teams and compare it with European professional football.^6–10^

## Material and methods

Two separate cohorts were included in the study, one including professional male football teams from South America and one including professional male football teams from Europe. In the South American cohort, six teams were included, all participating in the Copa Libertadores during the season that they were included. The South American cohort consisted of three Brazilian, two Argentinian teams and one team from Chile. For this cohort, data were collected between January and December of 2016.

In the European cohort, teams from the UEFA Elite Club Injury Study (ECIS) that qualified for the UEFA Champions League (UCL)-group stage for the 2015/2016 and 2016/2017 season were included. A total of 20 teams in ECIS qualified for UCL-group stage during 2015/2016, and their data from the 2015/2016 season were thus included in the present study. In the 2016/2017 season, a total of 23 teams from ECIS qualified for the UCL-group stage, and their data from the 2016/2017 season were also included. In total, 43 team-seasons (eight from England, seven from Spain, five from Portugal, four from France, four from Italy, three from Germany, two from Russia, two from the Netherlands, one from Belgium, one from Croatia, one from Denmark, one from Israel, one from Scotland, one from Switzerland, one from Turkey and one from Ukraine) were thus included in the European cohort for the present study.

### Patient involvement

This research was done without patient involvement. Patients were not invited to comment on the study design or contribute to this document’s writing or editing.

### Data recording

The same methodology was used to collect data in both the South American and the European cohorts and has been described earlier.^13^ At the beginning of the study, period clubs were asked to appoint a contact person (a member of the medical team) to be responsible for all data collection required for their participation in the study. Contact persons were given a study manual explaining the methodology and all operational definitions they needed to be aware of to register data uniformly. All football exposures, training and domestic league matches and international cup matches, were registered on an attendance record, including information about the duration of all football exposures on an individual player level. The attendance record also contained information about whether the exposures were from training sessions or matches.

In addition to the attendance records, contact persons were also asked to complete an injury for all time-loss injuries that occurred during football exposures. The injury form contained information about the injury occasion, for example, whether the injury occurred in training or during match-play and information about the injury mechanism. The injury form also contained information about the type and diagnosis of the reported injury. Table 1 describes the operational definitions that were used to collect data. Data were sent to the research group monthly and then controlled by the first author to ensure that all reported data were complete and following the study methodology. In case of any missing or unclear data, prompt feedback was sent back to the clubs to complete the reports.

**Table 1:** Operational definitions

Training session	Team training that involved physical activity under the supervision of the coaching staff
Match	Competitive or friendly match against another team.
Injury	Any physical complaint sustained by a player that resulted from a football match or football training led to the player being unable to participate in future football training or match play.
Ligament injury	Traumatic distraction injury to a ligament.
Muscle injury	Traumatic distraction or overuse injury to a muscle.
Injury incidence	The number of injuries per 1000 player hours ((Σ injuries/Σ exposure hours)×1000).
Injury burden	The number of lay-off days per 1000 player hours ((Σ lay-off days/Σ exposure hours)×1000).

### Statistical analyses

All statistical analyses were performed using the Statistical Package for the Social Sciences V.23. Injury incidence was defined as the number of injuries per 1000 match hours. Injury incidence (general injury incidence (including all injuries), muscle injury incidence and ligament injury incidence) were calculated for all football exposures as well as for training and match exposures specifically. Injury incidences are presented with 95% CIs. To compare injury incidences between the South American and the European cohort, rate ratios (RR) were calculated and presented with 95% CIs. RRs were tested for statistical significance using Z-statistics. Categorical variables were compared between cohorts using χ^2^-tests, while differences in quantitative variables were analysed with the Mann-Whitney U test. All tests were two-sided, and the significance level was set at p<0.05.

## Results

A total of 55 065 hours of exposure were registered in the South American cohort, including 49 699 (90%) training hours and 5366 (10%) match hours with an average of 857 training hours and 93 match hours per team and month. In the European cohort, a total of 307 721 hours were reported with 261 858 (85%) training hours and 45 863 (15%) match hours with an average of 561 training hours and 100 match hours per team and month.

The median training to match exposure ratio for the South American teams were 8.5 (IQR=7.9–11.5) and significantly higher (p=0.003) than the average training to match exposure ratio for teams in the European cohort (5.1 (IQR=4.4–6.8)).

### Injury patterns

A total of 271 injuries were reported in the South American cohort. In the European cohort, 1614 injuries were reported.

In the south American cohort, the majority of all reported injuries occurred during training. Training injuries thus constituted a significantly larger proportion of the reported injuries than in the European cohort. Muscle injuries and ligament injuries were the two most common injury types in both cohorts, with a significantly larger proportion of ligament injuries in the South American cohort (table 2).

**Table 2:** Comparison of injury characteristics between the South American and European cohorts

	South America	Europe	P value*
Injury occasion			
Training (n)	59% (159)	42% (681)	<0.001
Match (n)	41% (112)	58% (933)	<0.001
Injury type			
Muscle (n)	44% (120)	47% (761)	0.376
Ligament (n)	20% (55)	15% (247)	0.039
Other (n)	35% (96)	38% (605)	0.511
Injury mechanism			
Acute (n)	57% (155)	62% (1008)	0.097
Overuse (n)	43% (116)	38% (605)	0.097
Contact (n)	24% (64)	29% (461)	0.092
Non-contact (n)	76% (207)	71% (1152)	0.092
Reinjuries	10% (27)	8% (135)	0.388

*P values from χ^2^-tests comparing proportions of injury characteristics between cohorts.

The majority of all reported injuries were traumatic and non-contact in both cohorts. There was no significant difference in the proportion of traumatic-injury or overuse injury nor in contact or non-contact injuries between the two cohorts (table 2).

### General injury incidence

The injury incidence in the South American cohort was 4.9 injuries/1000 hours of exposure (95% CI 4.4 to 5.5). Training and match injury incidence for the South American teams are presented in table 3. The match injury incidence was more than six times higher than the training injury incidence (RR 6.52, 95% CI 5.12 to 8.31). There was no difference in the general or match injury incidences between the two cohorts. However, the training injury incidence was significantly higher in the South American cohort than the European cohort (table 4).

**Table 3:** Training and match injury incidences in the South American teams

	Injury incidence (95% CI)	
	Training	Match play
Team 1	2.0 (1.2 to 3.1)	7.8 (3.5 to 17.3)
Team 2	2.7 (1.5 to 4.8)	11.5 (5.2 to 25.6)
Team 3	3.2 (2.1 to 4.8)	18.6 (12.0 to 28.9)
Team 4	3.3 (2.4 to 4.5)	32.5 (23.3 to 45.3)
Team 5	3.8 (2.5 to 5.7)	27.3 (17.8 to 41.9)
Team 6	4.0 (2.9 to 5.4)	20.8 (13.9 to 31.0)

**Table 4:** Injury incidences and rate ratios between the South American and European cohorts

	Injury incidence (95% CI)		
	South America	Europe	Rate ratio (95% CI)
General injury incidence			
Training	3.2 (2.7 to 3.7)	2.6 (2.4 to 2.8)	1.23 (1.04 to 1.46)
Match	20.9 (17.3 to 25.1)	20.3 (19.1 to 21.7)	1.03 (0.84 to 1.25)
Total	4.9 (4.4 to 5.5)	5.2 (5.0 to 5.5)	0.94 (0.83 to 1.07)
Muscle injury incidence			
Training	1.4 (1.1 to 1.8)	1.3 (1.1 to 1.4)	1.10 (0.84 to 1.42)
Match	9.5 (7.2 to 12.5)	9.4 (8.5 to 10.3)	1.02 (0.76 to 1.36)
Total	2.2 (1.8 to 2.6)	2.5 (2.3 to 2.7)	0.88 (0.73 to 1.07)
Ligament injury incidence			
Training	0.6 (0.4 to 0.8)	0.3 (0.2 to 0.4)	1.87 (1.21 to 2.87)
Match	5.0 (3.5 to 7.3)	3.7 (3.1 to 4.3)	1.37 (0.91 to 2.06)
Total	1.0 (0.8 to 1.3)	0.8 (0.7 to 0.9)	1.24 (0.93 to 1.67)

### Muscle injury incidence

The muscle injury incidence in the South American cohort was 2.2 muscle injuries/1000 hours of exposure (95% CI 1.8 to 2.6). There were 69 muscle injuries reported in training and 51 in match-play. The match muscle injury incidence was thus close to seven times as high as the training injury incidence (RR 6.85, 95% CI 4.77 to 9.83). There were, however, no differences in total, training or match muscle injury incidences between the two cohorts (table 4).

### Ligament injury incidence

There were 61 ligament injuries reported in the South American cohort, 32 in training and 29 in match-play, representing a ligament injury incidence of 1.0 (95% CI 0.8 to 1.3). In match-play, the ligament injury incidence was almost eight times higher (RR 7.73, 95% CI 4.68 to 12.78) than in training. While there were no differences in total or match ligament injury incidences between the two cohorts, the training ligament injury incidence was significantly higher in the South American cohort than in the European cohort (table 4).

### Injury severity

The median period of absence following injuries in general among the South American teams were 9 days (IQR=4–18) in comparison with 11 (IQR=5–22) days in the European cohort. They showed a significant difference between cohorts (p=0.001). When specific injury types were analysed, a significant difference in lay-off between cohorts was found for muscle injuries (10 days (IQR=4–15) in the South American cohort compared with 13 days (IQR=7–20) in the European cohort, p=<0.001), whereas no difference between cohorts was found for ligament injuries (14 days (IQR=6–28) in the South American cohort compared with 16 days (IQR=7–32) in the European cohort, p=0.773). Analyses of proportions of different injury severities also showed significant differences between the two cohorts with more slight injuries in the South American cohort. When specific injury types were analysed separately, a significantly lower proportion of severe injuries and a higher proportion of slight injuries were found among muscle injuries in the South American cohort compared with the European cohort. In contrast, no differences between cohorts were found for proportions of injury severities of ligament injuries (table 5).

**Table 5:** Comparison of injury severity between the South American and European cohorts

	South America	Europe	P value*
All injuries			0.010
Slight (n)	21% (57)	14% (219)	
Minor (n)	22% (60)	22% (356)	
Moderate (n)	42% (114)	46% (740)	
Severe (n)	15% (40)	19% (299)	
Muscle injuries			0.002
Slight (n)	20% (24)	9% (72)	
Minor (n)	21% (25)	20% (154)	
Moderate (n)	53% (64)	58% (439)	
Severe (n)	6% (7)	13% (96)	
Ligament injuries			0.890
Slight (n)	11% (6)	9% (21)	
Minor (n)	20% (11)	20% (49)	
Moderate (n)	45% (25)	44% (108)	
Severe (n)	24% (13)	28% (69)	

*P values from χ^2^-tests comparing proportions of injury severities between cohorts for the specified injury type.

### Injury burden

There were no significant differences in injury burden in training or match play for all injuries, muscle injuries or ligament injuries between the two cohorts except for the ligament injury burden in training, which was significantly higher in the South American than in the European cohort (table 6).

**Table 6:** Comparison of injury burden between the South American and European cohorts

	Injury burden, median (IQR)		
	South America	Europe	P value*
General injury burden			
Training	51 (36–73)	50 (30–64)	0.703
Match	507 (144–585)	444 (275–598)	0.763
Total	86 (78–113)	114 (86–140)	0.215
Muscle injury burden			
Training	15 (8–18)	17 (7–28)	0.673
Match	117 (45–185)	141 (89–242)	0.321
Total	25 (10–37)	39 (18–61)	0.080
Ligament injury burden			
Training	10 (7–28)	4 (2–9)	0.017
Match	178 (71–328)	103 (31–205)	0.215
Total	32 (19–45)	21 (9–39)	0.185

*P values from Mann-Whitney U test comparing injury burden for the specified injury type between teams from the two cohorts.

## Discussion

The principal finding of the current study was that the injury panorama in the South American cohort had many similarities with the European cohort in the current study and with what has previously been reported from other studies.^10^ These similarities include that the injury incidence in matches was found to be several times higher than in training and that muscle injury was found to be the most common injury type.

### Higher ligament injury burden in South America

While several different injury variables were similar between South American and European football teams in the present study, ligament injuries during training were more common in South America than in Europe. This increased frequency also caused a higher ligament injury burden in the South American cohort even though the lay-off time following ligament injuries were similar in the two cohorts. A similarly high frequency of ligament injuries in South American football has previously been shown by Reis *et al*^11^ in a study following a Brazilian professional football team during one season during which 36% of the reported injuries affected joints and ligaments.

There are several plausible explanations for why ligament injuries were more frequent among the South American teams than in Europe. In Australian rules football, ligament injuries are more frequent in regions with a warmer climate.^8^ Similarly, in European football, the Anterior Cruciate Ligament injuries are more frequent in regions with a warmer climate.^7^ The climate in South America is typically warmer than in Europe. It is plausible that higher temperatures, and the differences in pitch conditions that this might cause,^7 8^ contributed to the increased ligament injury incidence among the South American teams.

### Are epidemiological time trends different between continents?

Europe has seen a decreasing trend in ligament injury incidence over the last two decades. Similar trends have been observed for specific diagnoses, such as ankle sprains and medial collateral ligament injuries^14 15^ and ligament injury incidence in general.^16^ It has been speculated that this decrease is an effect of successful implementation of preventive measures and changes in training philosophy, including more low-risk training sessions such as strengthening, conditioning and recovery sessions.^14–16^

Since no long-term analyses of injury patterns in South American professional football are available, one can only speculate if similar trends exist in South America. It could be argued that the difference in ligament injury incidence between the South American and European cohorts that were observed in this present study might indicate that there has not been a similar decreasing trend of the frequency of ligament injuries in South American football over the last decades.

### Are there differences in the training philosophy?

It is plausible that differences in training culture between South America and Europe could influence injury epidemiology. The South American teams in the present study were shown to have a higher training to match exposure ratio than their European counterparts, indicating that there is indeed a difference in training culture between the continents. The training injury incidence in the South American cohort was also higher than in the European cohort. In fact, due to a higher proportion of training exposure and a higher training injury incidence, the majority (59%) of all reported injuries from the South American cohort occurred in training. In comparison, the majority (58%) of all reported injuries from the European cohort occurred in match-play. The higher ligament injury incidence in the South American cohort was also observed in training. At the same time, no statistically significant differences were found in match play which may also indicate a difference in training culture between the two continents. Muscle injury incidence and proportions, on the other hand, were similar between cohorts in both training and match play which may be considered a sign that the content and physical demands of matches and training sessions were similar between the two continents. It should also be acknowledged that the present study’s findings are in contrast to what was observed in the study mentioned above by Reis *et al*.^11^ The proportion of training and match injuries was similar to the European cohort of the present study. To better understand why the frequency of some injuries differed between the two cohorts while others were similar, a larger sample size would be needed to allow for a more detailed analysis of injury diagnoses and injury mechanisms.^11^

### Methodological considerations

This study is strengthened by the fact that the methodology closely adheres to international consensus about how to conduct epidemiological research in football, making it easier to compare the results with previously published studies.^17 18^ To increase the reliability of data, close communication between the study group and the included clubs were continued throughout the study period. Data were sent to the study group monthly and were then reviewed by members of the study group to ensure that data were complete and following the study methodology. If any missing or uncertain data were identified in this review process, feedback was sent to the club to complete these uncertainties. In addition, two reports were sent to the participating clubs to increase the reliability of the reported data further.^17^

Some limitations of the study should, however, be acknowledged. A time-loss injury definition of injury was used in the present study. While time-loss is widely used to describe the severity of injuries in epidemiological studies, a time-loss definition of injury may underestimate the prevalence of overuse complaints that may not cause time-loss but could still severely hamper a players ability to perform on a football pitch.^17^

Unfortunately, no detailed information about the registered training sessions was available. While a difference in the ratio of training to match exposure hours was identified between the two continents, no data were available to describe the content of the training sessions in terms of intensity, drill selections, etc. Therefore, the hypothesis that a difference in training culture between the two continents could explain the differences in injury incidences remains speculative and needs to be investigated further.

The study is also limited by a relatively small sample size in the South American cohort with six included teams during one season. The generalisability of the results from these teams to the rest of the continent may thus be limited. It would also have been beneficial if the study had continued over several seasons, both to increase the sample size and also to identify possible time trends for important injury variables as has been done in European football. A large-scale and long-term injury surveillance study would be needed to understand the injury panorama in South American football fully.

## Data Availability

No data are available.
